# Gastric Kaposi sarcoma presenting as an upper gastrointestinal bleeding in a non-AIDS patient

**DOI:** 10.22088/cjim.12.0.413

**Published:** 2021

**Authors:** Elahe Zanganeh, Seyed Ashkan Hosseini, Mehdi Alimadadi, Mohammadreza Seyyedmajidi

**Affiliations:** 1 Golestan Research Center of Gastroenterology and Hepatology (GRCGH), Golestan University of Medical Sciences, Gorgan, Iran; 2 Mashhad University of Medical Sciences, Mashhad, Iran

**Keywords:** Kaposi sarcoma, Upper gastrointestinal bleeding, Non-AIDS

## Abstract

**Background:**

Majority of the patients affected by Kaposi sarcoma (KS) have human immunodeficiency virus (HIV). Highly active antiretroviral therapy (HAART) lessened the incidence of AIDS (acquired immunodeficiency syndrome)- related KS. Cutaneous signs are the most common, but involving the gastrointestinal (GI) tract is very important and dangerous because of serious complications including perforation and bleeding.

**Case Presentation:**

This report is a rare case of gastric-KS presenting as melena in a non-AIDS 67-year-old woman. We describe the diagnosis and management of this rare complication.

**Conclusion:**

GI-KS is often asymptomatic with different endoscopic appearances, and maybe present without cutaneous lesions. Thus, a high diagnostic suspicion is needed and we should attend these GI complications.

Moritz Kohn Kaposi described Kaposi sarcoma (KS) in 1872 as a low grade tumor of the vascular endothelium (1). KS is a rare type of cancer (0.001% of malignancies), but it was seen at a rate of 1000 times in human immunodeficiency virus (HIV)-infected patients (2). Although KS can develop at any stage of HIV infection, it occurs commonly in the setting of advanced immune suppression (3). 

The DNA of human herpesvirus 8 (HHV-8) has been detected in more than 95% of KS lesions as the causative agent (4). KS has four types (5,6): (a) Classic type: This type is in elderly Mediterranean men and involves the skin of lower extremities and rarely visceral organs with a benign course (b) Endemic type: It is seen in young African men and increases with age (c) Immunosuppression-related type: It occurs in patients with immunosuppressive therapy or organ transplant. This form is usually aggressive with visceral involvement. (d) AIDS-related type: It is the most common and aggressive form in the Western population. Up to half of the patients have visceral involvement (the gastrointestinal (GI) tract, lungs, liver, spleen, kidney and heart). The GI tract is the most commonly involved extra-cutaneous site and GI lesions can be present without cutaneous lesions (7-10). 

The majority are clinically silent and most visceral KS remain unidentified. Therefore, some experts have suggested screening endoscopy for the early detection and initiation of treatment to improve survival outcome (11). We present an uncommon case of non-AIDS-related KS complicated by upper GI bleeding. This problem describes the need for awareness of GI complications of KS.

## Case presentation

**Figure 1:**
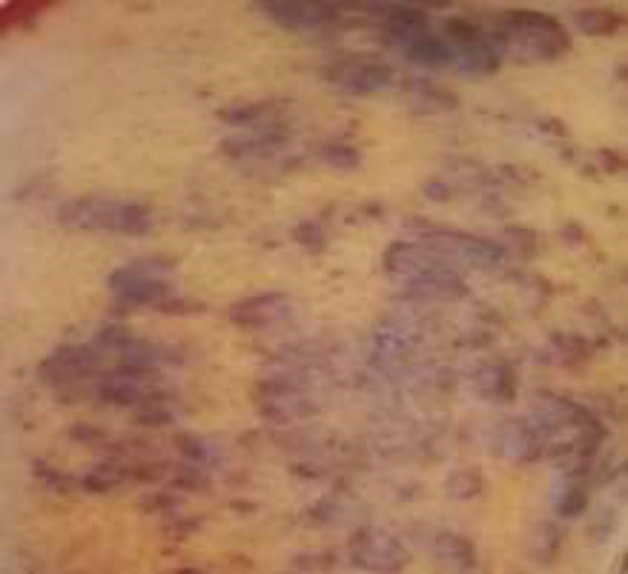
Multiple, plaque-like, violaceous skin lesions in lower extremities

A 67-year-old woman presented our hospital with complaints of pallor and weakness for a few days. She had a mild abdominal pain and black tarry stool. Her past medical history was not significant. The patient was well nourished with multiple, plaque-like, violaceous skin lesions increasing in both lower extremities since last year (fig. 1). Physical examination revealed anemia and epigastric tenderness. Laboratory analyses showed white blood cell: 7,900; hemoglobin: 9.6 g/dL; MCV: 74 fL; platelet count: 267,000; Biochemistry analysis revealed normal creatinine, plasma sugar, amylase, liver enzymes, bilirubin, erythrocyte sedimentation rate (ESR) and C-reactive protein. Serum polymerase chain reaction (PCR) test for HIV was negative. 

**Figure 2:**
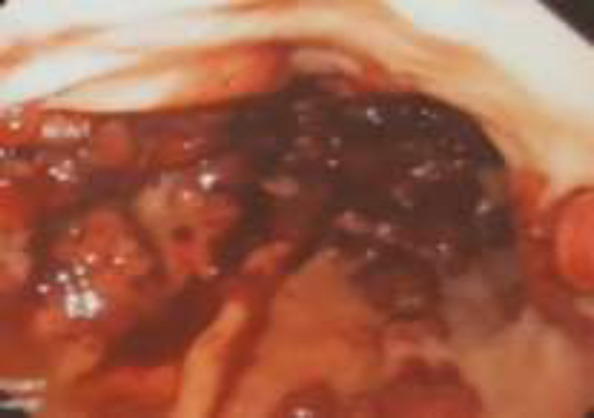
Gastric polypoid/nodular KS lesions

**Figure 3:**
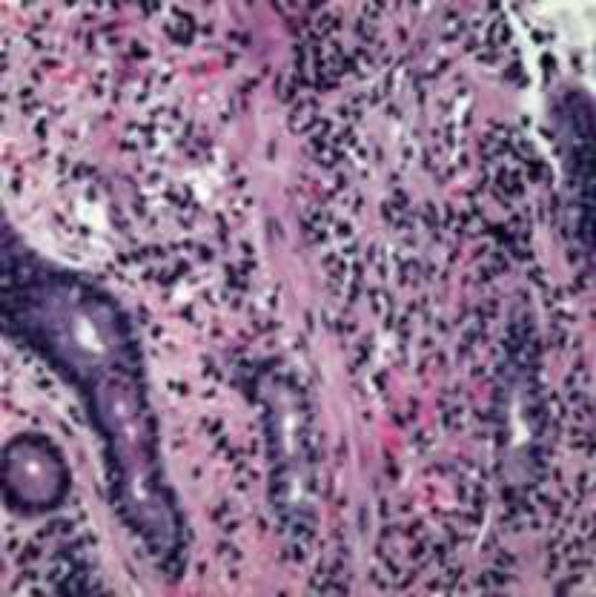
Spindle cell proliferation and red blood cell extravasation with extensive lymphoplasmocytic cell infiltration in hematoxylin and eosin stain

**Figure 4:**
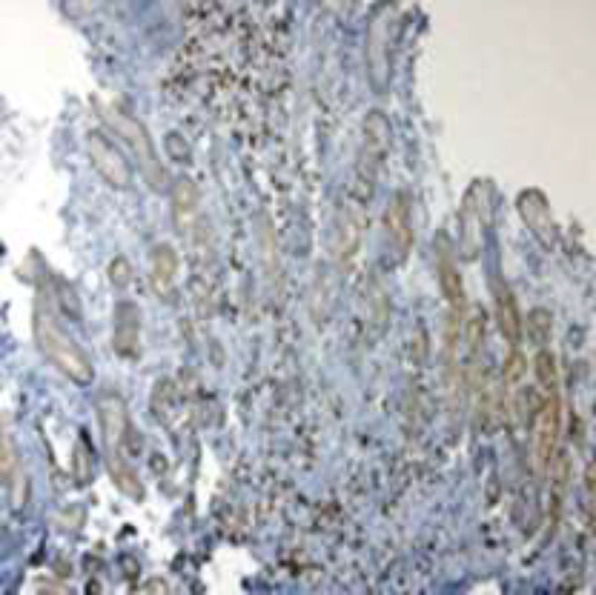
Positive Immunohistochemical test for HHV-8

Upper GI endoscopy revealed multiple friable polypoid and nodular lesions of varying sizes in the stomach (fig. 2). Endoscopic biopsy specimens were obtained and hematoxylin and eosin examination showed high vascularity, spindle cells proliferation with extensive lymphoplasmocytic cell infiltrates. Immunohistochemical test was positive for HHV-8 supporting the diagnosis of gastric KS (figs. 3, 4). Hydration, supportive care and argon plasma coagulation (APC) during endoscopy for oozing lesions were performed. The patient was discharged and referred to oncology clinics.

## Discussion

The diagnosis of KS in the gut should be considered in elderly men from Eastern Europe and Mediterranean regions and specially in immunosuppressed and AIDS patients. Greater-than-50% is the incidence of GI-KS is in AIDS patients with cutaneous KS (9). Although it is usually asymptomatic, bleeding from the oral cavity, esophagus, stomach, and bowel have been reported. Further complications are perforation or obstruction (12, 13). Non-Hodgkin lymphoma, as a differential diagnosis, frequently involves GI tract in AIDS patients. Furthermore, tumors of GI tract with spindle-shaped cells such as leiomyoma, rhabdomyosarcoma or gastrointestinal stromal tumors (GISTs) have to be considered in the differential diagnosis (14).

The diagnosis of KS with a negative HIV test should lead to the consideration of other causes such as iatrogenic or lymphoproliferative disorders-related immunosuppression (9). Iatrogenic KS among kidney recipients is more than the other solid organ recipients. Among these patients, KS is more detected in HHV-8 endemic areas. It typically occurs within the first two years after the start of immunosuppression (15-17). Over 95% of lesions, regardless of clinical subtype, have been infected with HHV-8. The long-lasting expression of HHV-8 genes is important for spindle-cell progression (18).

GI appearances in endoscopy are variable as flat ulcerated, polypoid/nodular, and volcano-shaped lesions. Biopsy is the gold diagnostic test of both the cutaneous and GI lesions. In Histology, they have typical features and morphology in hematoxylin and eosin staining (7). If it is not sufficient to diagnosis, immunohistochemical studies can help us. The antibody of latent nuclear antigen-1 (LAN-1) encoded by HHV-8 has been available since 2002 and has excellent sensitivity and specificity for KS (19).

Therapy depends on the clinical subtype and organ involvement and it is usually palliative and aimed at improving symptoms and preventing progression. For visceral involvement and AIDS-associated KS, chemotherapy combined with highly active antiretroviral therapy (HAART) may be indicated (7). In patients with immunosuppression, reduction or withdrawal of immunosuppressant is advised. Adoption of HAART in the treatment of HIV decreased the incidence of KS but a high suspicion in susceptible patients increases the likelihood of early diagnosis and management of the aggressive disease. Systemic chemotherapy is usually reserved for cases with widespread disease (7). Overall, the visceral involvement of KS has poor prognosis, with a 6-month survival of 40% (20). One study suggested endoscopy in the patients with cutaneous lesions, CD4+ T-cell count less than100/μL and men who have sex with other men (21). We reported a rare case of gastric-KS with cutaneous lesions presenting as melena in a non- immunosuppressed elderly woman in Iran.

In conclusion, GI-KS is often asymptomatic with different endoscopic appearances, and maybe present without cutaneous lesions. Thus, a high diagnostic suspicion is needed and we should attend these GI complications.
